# Impact of educational training and C-reactive protein point-of-care testing on antibiotic prescribing in rural and urban family physician practices in Latvia: a randomised controlled intervention study

**DOI:** 10.1186/s12887-022-03608-4

**Published:** 2022-09-21

**Authors:** Zane Likopa, Anda Kivite-Urtane, Vija Silina, Jana Pavare

**Affiliations:** 1 Children’s Clinical University Hospital, Vienibas Gatve 45, Riga, LV-1004 Latvia; 2 Riga Stradins University, Dzirciema 16, Riga, LV-1007 Latvia; 3 Department of Public Health and Epidemiology, Institute of Public Health, Riga Stradins University, Kronvalda boulevard 9, Riga, LV-1010 Latvia; 4 Department of Family Medicine, Riga Stradins University, Anninmuizas boulevard 26a, Riga, LV-1067 Latvia

**Keywords:** Acute infections, Children, Antibiotic prescription, Primary care, Point-of-care testing, Education

## Abstract

**Background:**

Although self-limiting viral infections are predominant, children with acute infections are often prescribed antibiotics by family physicians. The aim of the study is to evaluate the impact of two interventions, namely C-reactive protein point-of-care testing and educational training, on antibiotic prescribing by family physicians.

**Methods:**

This randomised controlled intervention study included acutely ill children consulted by 80 family physicians from urban and rural practices in Latvia. The family physicians were divided into two groups of 40. The family physicians in the intervention group received both interventions, i.e. C-reactive protein point-of-care testing and educational training, whereas the family physicians in the control group continued to dispense their standard care. The primary outcome measure was the antibiotic prescribing at the index consultation (delayed or immediate prescription) in both study groups. The secondary outcome was CRP testing per study group. Patient- and family physician- related predictors of antibiotic prescribing were analysed as associated independent variables. Practice location effect on the outcomes was specially addressed, similar to other scientific literature.

**Results:**

In total, 2039 children with acute infections were enrolled in the study. The most common infections observed were upper and lower respiratory tract infections. Overall, 29.8% (*n* = 607) of the study population received antibiotic prescription. Our binary logistic regression analysis did not find a statistically significant association between antibiotic prescriptions and the implemented interventions. In the control group of family physicians, a rural location was associated with more frequent antibiotic prescribing and minimal use of CRP testing of venous blood samples. However, in the intervention group of family physicians, a rural location was associated with a higher level of C-reactive protein point-of-care testing. Furthermore, in rural areas, a significant reduction in antibiotic prescribing was observed in the intervention group compared with the control group (29.0% (*n* = 118) and 37.8% (*n* = 128), respectively, *p* = 0.01).

**Conclusion:**

Our results show that the availabilty of C-reactive protein point-of-care testing and educational training for family physicians did not reduce antibiotic prescribing. Nevertheless, our data indicate that regional variations in antibiotic-prescribing habits exist and the implemented interventions had an effect on family physicians practices in rural areas.

## Introduction

Acute infection in children is one of the most common reasons for attending family physicians (FP) and these visits often result in antibiotic prescription. The most frequent indication for antibiotic use is respiratory infection, with the highest incidence rate for very young children (up to 2 years old) [1] despite viral aetiology predominance in this age group that does not require specific treatment [2]. Indeed, at least 30% of antibiotics prescribed in out-patient settings are considered to be unnecessary [3].

Recent studies have shown that inappropriate antibiotic usage may be due to several reasons. These include difficulties differentiating between viral and bacterial diseases based on clinical signs alone [4, 5], fear of complications or missing serious bacterial infections, heavy workloads and even parental insistence on antibiotics being prescribed [3]. In order to optimise outpatient antibiotic prescribing, the effectiveness of several different types of interventions has been assessed. These include patient and FP education, communication training, point-of-care testing (POCT), active prescription monitoring and delayed prescribing [3]. However, as a multidirectional combination of interventions is more likely to reduce unnecessary antibiotic prescribing than a single intervention [3, 6, 7], we conducted the present study to evaluate the effect of two interventions, namely access to C-reactive protein (CRP) POCT in FP practices and educational training for FP, focused on targeted antibiotic prescribing.

CRP is an acute phase protein that can reduce diagnostic uncertainty. In recent years, it has been more widely used for POCT in routine primary care practice in several European countries [8]. The main advantages of CRP POCT are its ease of use, rapid feedback of the test result allowing immediate decision-making on whether or not to prescribe antibiotics and higher patient satisfaction with the child-friendly finger prick test instead of an invasive venous puncture [9]. In Latvia, CRP POCT is currently available in only a few FP practices and not state covered. In the main, venous blood samples are sent to laboratories for CRP detection. However, especially in rural areas of Latvia, the result might not be reported until the next working day, which is often too long a delay for acutely ill patients and consequently the decision to initiate antibacterial treatment is frequently based upon clinical examination alone. A previous meta-analysis has shown that CRP POCT is associated with a lower antibiotic prescription rate for adults with respiratory tract infections in primary care [10]. However, the findings regarding CRP POCT for children with acute illnesses are presently incomplete and controversial and thus this subject requires further analysis [11]. Additionally, interventions such as patient, parent and physician education and communication training have also been shown to have an impact on reducing antibiotic prescribing [12]. However, data on impact of such interventions on antibiotic prescription specifically by FPs is scarce.

In this study as the second intervention, we included educational training of FP s based on new recommendations for the management of respiratory infections as well as fever for children that have been recently introduced in Latvia. Furthermore, although the Happy Audit study has previously reported that interventions focused on patient or FP education and CRP POCT for adults reduce inappropriate antibiotic prescriptions [13–15], the effectiveness of these two types of interventions on antibiotic prescribing has not been evaluated in a paediatric population.

## Materials and methods

This randomised controlled intervention study was conducted in Latvia between November 2019 and February 2020. The aim of the study was to evaluate the effect of the combination of two interventions – access to CRP POCT in FP practices and educational training for FP – on the antibiotic prescribing rate of FP for acutely ill children. Patient- and FP-related predictors of antibiotic prescribing were also analysed.

### Participating family physicians

There are approximately 360,000 children (age 0–17 years) in Latvia and their health needs are served by an estimated 1268 FP. FP are self-employed, usually independently located and serve an extensive age range of children. For the purposes of this study, we had access to 40 CRP POCT devices, enabling 80 FP to be recruited. The FP selection process has previously been reported [16]. Briefly, the participating FP were recruited using two approaches. First, from the country’s 1268 FP, by means of an Excel random-number generator, we selected 160 FP (the expected response rate was 50%) across different geographically located practices (urban and rural areas) and sent invitations to participate in the study via both email and paper-based letter form. Unfortunately, the response rate was lower than expected and only 38 participants were recruited using this approach. Secondly, we directly addressed FP at a meeting of the Latvian Family Physicians Association and consequently achieved the requisite number of 80 participants. It was expected that each FP might see about 30 suitable patients during the duration of the study.

### Interventions

FP were stratified according to practice location and each stratum was divided into two groups of 40 FP using random numbers generated by MS Excel Random Number function. Practices in the intervention group received both interventions (CRP POCT and educational training). Specifically, each FP in this group received a CRP POCT device for use during the duration of the study and was individually tutored by diagnostic test company on how to perform the CRP test during a face-to-face meeting, and ongoing support by the company was available to the FP. In addition, FP were contacted proactively by the study team to address any issues. We used the Orion Diagnostica QuikRead go CRP POCT system for the quantitative determination of CRP in blood with a sample volume of 20 μl obtained via a finger prick. This system has a measuring range of 5–200 mg/L and the result is available within 2 minutes. As CRP cut-off levels for children in primary care are currently undetermined [17], the FP did not receive any guidelines on the interpretation of results. FP in the intervention group were allowed to order a CRP test based on individual indications if they believed the result would help them make a more informed decision on antibiotic necessity after a clinical assessment. Other tests such as rapid strep test, urine dipstick test or laboratory analyses were also available as usual; however, the availability of testing was different between urban and rural practices.

FP in the intervention group also received educational training based on new recommendations for the management of respiratory infections and fever in children introduced in 2019 in Latvia. The key topics were:

- child with fever – evaluation, precautionary level system and management,
- child with upper and lower respiratory infection – evaluation and management,
- principles of antibiotic resistance and safer prescribing of antibiotics.

This intervention involved one four-hour training seminar, followed by educational materials in video and printed format. FP also received parent information booklets about managing children with fever at home and signs to look out for that indicate a FP should be contacted.

FP in the control group received no interventions and continued to dispense their standard care.

### Participating children

FP were asked to record the data of consecutive children (1 month up to 17 years old) who attended their practice for a face-to-face visit with current clinical signs of an acute infection that had been present for less than 5 days. Patients were excluded if they were aged under 1 month, had received antibiotics prior to the visit or were already in the reconvalescent stage of disease.

### Sample size

We presumed that the frequency of antibiotic prescribing in the intervention group compared to the control group was 34 and 42%, respectively, as per Martínez-González et al. [18]. Thus, according to Fleiss et al. [19], with 80% power and α level 5%, our study required 571 patients in each study group.

### Data collection

FP collected data in anonymised form. The variables recorded included whether or not an antibiotic was prescribed, patient demographics, and diagnosis based on a pre-defined list (upper respiratory infections (common cold, rhinosinusitis, otitis, pharyngitis, tonsillitis, stomatitis, laryngitis), lower respiratory infections (bronchitis, bronchiolitis, pneumonia), gastrointestinal infections, urinary tract infections, skin and soft tissue infections and joint and bone infections) and the diagnostic tests undertaken prior to the initiation of antibiotic treatment (e.g. CRP POCT, CRP measurement from a venous blood sample, full blood count, urine dipstick test and microscopy, *Group* A streptococcal *rapid antigen test*, rapid influenza diagnostic test, bacteriological cultures, X-ray).

The primary outcome measure was the antibiotic prescribing at the index consultation (delayed or immediate prescription) in both study groups. The secondary outcome was CRP testing per study group. Patient- and family physician- related predictors of antibiotic prescribing were analysed as associated independent variables. Practice location effect on the outcomes was specially addressed, similar to other scientific literature.

### Statistical analyses

Descriptive statistics, such as means (with standard deviations) and medians (with interquartile range (IQR)) for continuous variables and proportions for categorical variables, were calculated. For determination of the statistical significance of differences in the proportions of dependent variables between subgroups of independent variables, the Chi-square test was used. Normal distribution was tested using the Kolmogorov-Smirnov test. To identify factors associated with antibiotic prescription or CRP testing, binary logistic regression was used.

Results were considered as statistically significant if *p* < 0.05. Data processing was performed using IBM SPSS Statistics (Statistical Package for the Social Sciences, Version 23.0).

## Results

Initially, 80 FP started the study; however, 5 FP from the control group declined to participate further after randomisation. The mean age of the FP was 51.9 years and the majority were female. Considerable heterogeneity existed regarding the length of time working in FP practice (ranging from 1 year to 52 years) and the number of paediatric patients registered at practices (ranging from 48 to 1843 children). Table 1 details the characteristics of the FP in the intervention and control groups. There were no significant differences between the two groups regarding the age, sex and work experience of the FP, number of registered paediatric patients and practice location.

**Table 1:** Characteristics of family physicians according to the study groups

Variables	Intervention group (***n*** = 40)	Control group (***n*** = 35)
Age (years)		
Median	52.5 (IQR 46.3–59.8)	53.0 (IQR 46.0–61.0)
Sex		
Male	1 (2.5%)	1 (2.9%)
Female	39 (97.5%)	34 (97.1%)
Work experience (years)		
Mean	25.4 (SD 13.1)	24.6 (SD 11.9)
Proportion of children on patient list (%)		
Median	24.3 (IQR 16.7–43.4)	24.2 (IQR 16.9–38.1)
Location		
Rural areas	14 (35.0%)	10 (28.6%)
Regional cities	10 (25.0%)	8 (22.9%)
Capital of Latvia	16 (40.0%)	17 (48.6%)

**Fig. 1:**
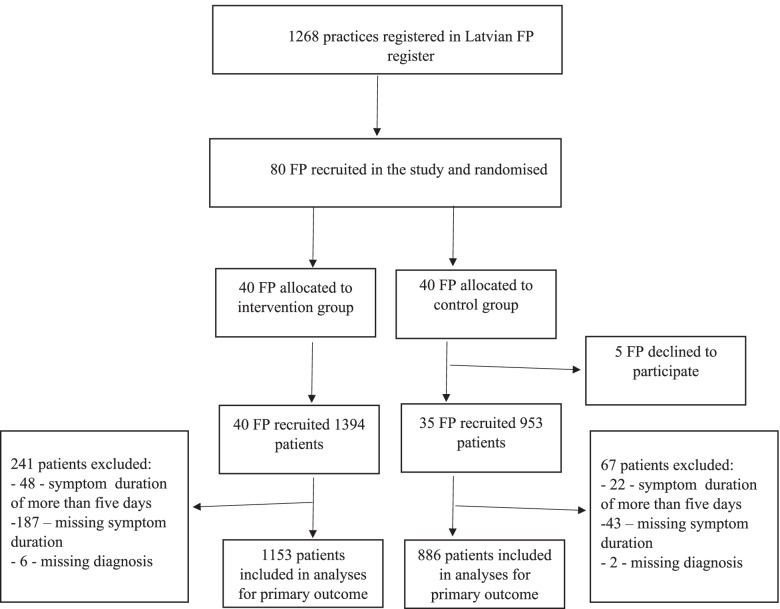
Flowchart of the study’s recruitment process. FP: family physician

During the three-month study period, 2347 patients were recruited; however, 308 patients were excluded due to symptom duration of more than 5 days or missing information concerning diagnoses (Fig. 1). Therefore, a total of 2039 patients met the inclusion criteria (1153 patients in the intervention group and 886 in the control group). The mean number of included patients per FP was 27.2.

The mean age of the patients was 6.1 years. Boys comprised 50.9% of the study participants. The mean duration of illness was 2.6 days. Only 8.7% of patients had a chronic disease and bronchial asthma was the most common (84.8%). Table 2 summarises the characteristics of the patients in both study groups. There were small imbalances between the groups regarding age, duration of symptoms, frequency of chronic disease and vaccination status.

**Table 2:** Characteristics of patients according to the study groups

Variables	Intervention group (***n*** = 1153)	Control group (***n*** = 886)
Age (years)		
Median	5.0 (IQR 3.0–9.0)	5.0 (IQR 2.0–8.0)
0–4 years	484 (42.6%)	431 (49.4%)
5–9 years	383 (33.7%)	279 (32.0%)
10–14 years	186 (16.4%)	120 (13.8%)
15–17 years	82 (7.2%)	42 (4.8%)
Sex		
Boys	591 (51.6%)	440 (50.0%)
Girls	555 (48.4%)	440 (50.0%)
Duration of illness (days)		
Median	3.0 (IQR 2.0–4.0)	3.0 (IQR 2.0–4.0)
Chronic disease		
Yes	75 (6.5%)	102 (11.5%)
No	1078 (93.5%)	784 (88.5%)
Full vaccination	1046 (92.7%)	820 (95.0%)
Partial vaccination	69 (6.1%)	40 (4.6%)
No vaccination	13 (1.2%)	3 (0.3%)
Diagnoses		
Upper respiratory infection	922 (80.0%)	675 (76.2%)
Lower respiratory infection	204 (17.7%)	180 (20.3%)
Gastrointestinal infection	17 (1.5%)	19 (2.1%)
Urinary tract infection	8 (0.7%)	10 (1.1%)
Skin and soft tissue infection	2 (0.2%)	1 (0.1%)
Bone and joint infection	0	1 (0.1%)
Ambulatory patients	1136 (98.5%)	879 (99.2%)
Referred to hospital	17 (1.5%)	7 (0.8%)

^a^Denominators may vary due to the missing values

No significant difference was found in the distribution of diagnoses between the two groups. The most common infections were upper respiratory tract infections (78.3% (*n* = 1597) of patients) and lower respiratory tract infections (18.8% (*n* = 384)). Gastrointestinal (1.8% (*n* = 36)), urinary tract (0.9% (*n* = 18)), skin and soft tissue (0.1% (*n* = 3)), and bone and joint (0.05% (*n* = 1)) infections featured to a much lesser extent.

**Fig. 2:**
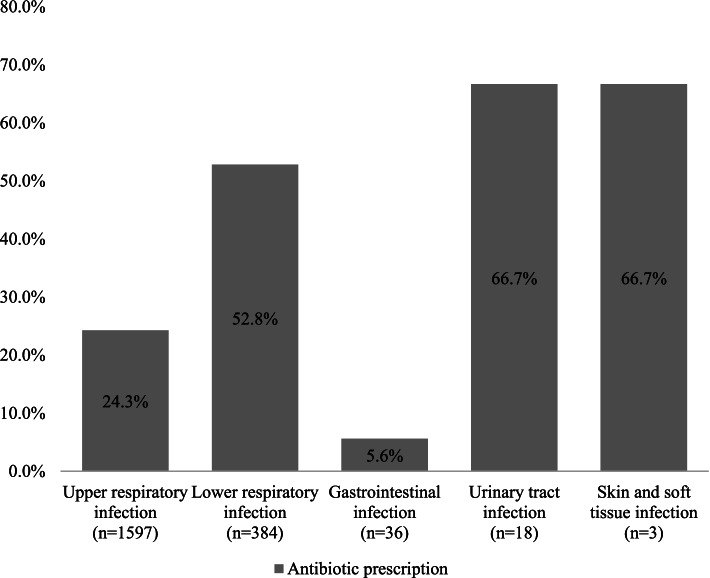
Proportion of all patients (%) treated with antibiotics for each type of infection

Overall, 29.8% (*n* = 607) of the study population received antibiotic prescription. The proportions of patients treated with antibiotics for each type of infection are shown in Fig. 2.

Comparing the two study groups, 27.8% (*n* = 320) episodes prompted antibiotic prescription in the intervention group, whereas it was 32.4% (*n* = 287) in the control group. This difference was statistically significant (*p* < 0.02).

Table 3 shows the patient- and FP-related predictors of antibiotic prescribing, also including the interventions as a single factor. Our data showed that antibiotic prescribing was significantly associated with younger children (adjusted odds ratio (aOR) for children aged 10–14 years vs. 0–4 years was 0.62, *p* = 0.002), middle-aged FP (aOR for FP aged 41–50 years vs. 30–40 years was 1.76, *p* = 0.002), a rural location of the FP practice (aOR was 1.42, *p* = 0.01 compared to the capital city) and a larger number of registered paediatric patients (aOR was 1.68, *p* < 0.001 and 1.59, *p* = 0.02 for 501–1000 and 1001+ patients, respectively, compared to < 500 patients).

**Table 3:** Patient- and FP-related predictors of antibiotic prescribing (as per unadjusted analysis and binary logistic regression model)

Characteristics	Antibiotic prescriptions n (%)	Crude OR (95% CI)	***P***	Adjusted OR^a^ (95% CI)	***P***
**Patient-related factors**					
Age (years)					
0–4	294 (32.1)	1		1	
5–9	187 (28.2)	0.83 (0.67–1.04)	0.10	0.81 (0.65–1.02)	0.07
10–14	71 (23.2)	**0.64 (0.47–0.86)**	**0.003**	**0.62 (0.46–0.85)**	**0.002**
15–17	42 (33.9)	1.08 (0.73–1.61)	0.70	1.10 (0.73–1.66)	0.64
Sex					
Boys	295 (28.6)	1		1	
Girls	309 (31.3)	1.12 (0.93–1.36)	0.23	1.16 (0.96–1.41)	0.14
Duration of symptoms (days)					
1	22 (22.2)	1		1	
2	152 (25.0)	1.17 (0.70–1.94)	0.55	1.08 (0.64–1.81)	0.78
3	223 (33.1)	**1.73 (1.05–2.85)**	**0.03**	1.53 (0.91–2.57)	0.11
4	137 (32.6)	**1.69 (1.01–2.84)**	**0.045**	1.57 (0.92–2.68)	0.10
5	73 (30.5)	1.54 (0.89–2.66)	0.12	1.50 (0.84–2.65)	0.17
**FP-related factors**					
Age (years)					
30–40	89 (25.2)	1		1	
41–50	172 (36.1)	**1.67 (1.23–2.27)**	**0.001**	**1.76 (1.24–2.51)**	**0.002**
51–60	167 (25.7)	1.02 (0.76–1.38)	0.88	0.96 (0.69–1.32)	0.79
61+	179 (32.1)	**1.40 (1.04–1.89)**	**0.03**	1.12 (0.80–1.57)	0.52
Sex					
Male	15 (32.6)	1		1	
Female	529 (29.7)	0.87 (0.47–1.63)	0.67	0.92 (0.47–1.80)	0.81
Work experience					
< 5 years	48 (25.7)	1		1	
6–10 years	15 (14.6)	**0.49 (0.26–0.94)**	**0.03**	0.70 (0.35–1.41)	0.32
11–20 years	106 (31.4)	1.32 (0.89–1.98)	0.17	1.52 (0.96–2.41)	0.08
21+ years	438 (31.0)	1.30 (0.92–1.84)	0.13	1.28 (0.87–1.86)	0.21
Location of practice					
Rural areas	246 (33.0)	**1.28 (1.04–1.59)**	**0.02**	**1.42 (1.10–1.84)**	**0.01**
Regional cities	119 (28.3)	1.03 (0.80–1.34)	0.82	1.23 (0.92–1.65)	0.16
Capital of Latvia	242 (27.7)	1		1	
Number of paediatric patients in practice					
< 500	266 (25.6)	1		1	
501–1000	267 (34.7)	**1.54 (1.26–1.89)**	**< 0.001**	**1.68 (1.33–2.11)**	**< 0.001**
1001+	74 (31.9)	1.36 (1.26–1.89)	0.05	**1.59 (1.09–2.34)**	**0.02**
Study group					
Intervention	320 (27.8)	**0.80 (0.66–0.97)**	**0.02**	0.83 (0.67–1.03)	0.09
Control	287 (32.4)	1		**1**	

^a^Adjusted OR: adjusted odds ratio – adjusted for all independent variables in the table, except the age of FP due to the collinearity with the duration of the career of FP (for age of FP variable adjustment has been carried out for all the variables except the duration of career)

Our binary logistic regression analysis did not find a statistically significant association between antibiotic prescriptions and the implemented interventions. However, subgroup analysis by location of practice showed a significant reduction in antibiotic prescribing in the intervention group compared to the control group in rural areas (29.0% (*n* = 118) and 37.8% (*n* = 128), respectively, *p* = 0.01). The proportions of antibiotic prescriptions in relation to location of practice in both study groups are presented in Table 4.

**Table 4:** Antibiotic prescribing according to FP practice location in the two groups

Location of practice	Antibiotic prescriptions, n (%)		
	Intervention group	Control group	***P***
Rural areas	118 (29.0)	128 (37.8)	**0.01**
Regional cities	69 (26.1)	50 (32.1)	0.19
Capital of Latvia	133 (27.6)	109 (27.9)	0.93

The CRP level was frequently measured in the intervention group; 72.4% (*n* = 835) of episodes, with CRP POCT being preferred over standard laboratory testing of a venous blood sample (99.0% (*n* = 827) and 1.0% (*n* = 8), respectively). For 8 patients with CRP POCT testing of a venous blood sample was also provided. In contrast, the CRP level was measured in just 8.8% (*n* = 78) of episodes in the control group, where only venous blood sample testing was available. Furthermore, 79.4% of antibiotic prescriptions were preceded by CRP testing in the intervention group compared with only 12.5% in the control group. Our binary logistic regression analysis found a significant association between higher usage of CRP testing and rural location of FP practice in the intervention group (aOR was 2.51, *p* < 0.001). However, the relationship in the control group was the opposite, with patients more likely to have their CRP level tested in laboratories in urban areas than in rural areas (aOR was 0.05, *p* < 0.001) (Table 5).

**Table 5:** Binary logistic regression analysis of the effect of location of the FP practice on CRP testing in the two groups

Intervention group			Control group			
Characteristic	CRP testing n (%)	Adjusted OR^a^ (95% CI)	***P***	CRP testing n (%)	Adjusted OR^a^ (95% CI)	***P***
Location of practice						
Rural areas	370 (90.9)	**2.51 (1.57–4.00)**	**< 0.001**	3 (0.9)	**0.05 (0.02–0.17)**	**< 0.001**
Regional cities	145 (54.9)	**0.39 (0.27–0.57)**	**< 0.001**	20 (12.8)	0.99 (0.53–1.86)	0.99
Capital of Latvia	328 (68.0)	1		55 (14.1)	1	

^a^Adjusted OR: adjusted odds ratio – adjusted for patient-related factors (age, sex, duration of symptoms) and FP-related factors (age, sex, work experience, number of registered paediatric patients), except the age of FP due to the collinearity with the duration of the career of FP

The majority of patients who underwent CRP testing – 78.9% (*n* = 727) – were found to have a CRP level < 20 mg/L, whereas 15.3% (*n* = 141) had a level between 20.01 and 50 mg/L, 4.5% (*n* = 41) between 50.1 and 99 mg/L, and 1.3% (*n* = 12) > 100 mg/L. Of note, 28.4% (*n* = 317) of all patients who did not undergo CRP testing were prescribed antibiotics by FP. Furthermore, of the patients with a CRP level < 20 mg/L, 19.9% also received antibiotics. Overall, antibiotic prescribing increased with increasing measured CRP level (*p* < 0.0001).

## Discussion

The aim of this study was to evaluate the effect of CRP POCT in FP practices and educational training for FP on antibiotic prescribing for children in primary care – a two-intervention combination that has not previously been studied in a paediatric population. CRP POCT is already routinely used in primary care by several other countries [8]. A meta-analysis has previously shown that CRP POCT significantly reduces immediate antibiotic prescribing for adults compared with standard care [18]; however, evidence for the use of CRP POCT to guide antibiotic prescribing for children is currently lacking. Nonetheless, a reduction in antibiotic prescribing for children has been reported in studies in which guidance for CRP result interpretation was provided [20–22]. Educational training of primary health care providers or parents on appropriate antibiotic usage has been shown to be effective in changing the antibiotic-prescribing behaviour [23, 24]. Multifaceted interventions, including educational training for physicians and patients, communication skills training and the introduction of POCT into clinical practice, have the potential to reduce inappropriate antibiotic usage even further [7, 15].

Although we did not find a statistically significant association between antibiotic prescriptions and the implemented interventions in regression analysis, analysing the data by location of practice revealed that the interventions significantly reduced antibiotic usage in rural areas (29.0% in the intervention group versus 37.8% in the control group). To the best of our knowledge, this is the first time the effectiveness of CRP POCT and educational training on antibiotic prescribing has been analysed by practice location in a paediatric population.

The overall effect was smaller than anticipated when comparing the intervention and control groups; however, this should be seen in the light of an overall low antibiotic prescribing rate. Other studies have pointed out that interventions may be more beneficial in cases of generally higher prescribing [25]. The observed antibiotic prescribing rate was lower than expected in both study groups (27.8% in the intervention group and 32.4% in the control group). Dumpis et al. previously reported an antibiotic prescribing rate of 42% in Latvia and 53% in Lithuania [26]. It is possible that during the study period our recruited FP were more inclined to avoid prescribing antibiotics. However, despite a low general antibiotic prescribing rate, significant variations were detected between rural and urban areas. Higher overall antibiotic prescribing rates were associated with practices located in rural areas (aOR was 1.42, *p* = 0.01) compared with the capital city. This finding is consistent with those of previous studies where rurality was found to be a risk factor for inappropriate and more frequent prescribing [3, 27, 28]. More extensive use of antibiotics in rural areas may be explained by fewer FP per head of population, increased workload, limited access to laboratory testing with consequent diagnostic uncertainty [28] and fears of missing secondary bacterial infections which may occur when patients are unable to access medical care [29].

We observed a markedly higher level of CRP testing in the intervention group (72.4% compared to the control group (8.8%). Furthermore, antibiotic prescriptions were preceded by CRP testing far more frequently in the intervention group. Moreover, in line with previous studies [9], FP in the intervention group almost exclusively used POCT (99.0%) for CRP level measurement rather than laboratory testing of a venous blood sample. This highlights user friendliness, patient satisfaction and clinical utility as the main advantages of CRP POCT. The majority of FP did not have any previous experience of using CRP POCT and so perhaps were more interested in trying it during the study period, thus resulting in the observed high usage level in the intervention group. Having said that, other studies have reported that CRP testing is also widespread for self-limiting viral infections in countries where POCT is available [30]. For comparison, in Sweden the level of CRP is measured in up to 50% of all consultations for respiratory infections [25]. The very low CRP testing level in the control group, especially in rural areas, may be due to differences in the availability of testing and timing of reporting of test results from central laboratories.

Although we observed a low rate of antibiotic prescribing, unnecessary antibiotic prescribing still occurs. Antibiotics were often prescribed at the early stage of disease as we only included patients with a symptom duration of less than 5 days (median duration 3 days). Overall, the duration of symptoms was not associated with higher antibiotic prescribing. We found that antibiotics were still widely used for self-limiting upper respiratory tract infections (26.0% in the control group and 23.1% in the intervention group), despite the majority of tested patients having a low CRP concentration and consequently not requiring antibacterial treatment. Moreover, for 19.9% of patients with a CRP level < 20 mg/L, antibiotics were prescribed. These data indicate that FP require more experience and guidance interpreting CRP level results.

### Strengths and limitations

The FP response rate was lower than expected from random selection and it is possible that the participating FP may have been more active and inclined to perform well. Nevertheless, our study included FP with different paediatric patient counts, work experience and practice localities, thus providing widespread information about the country. Moreover, as CRP POCT is not currently integrated into primary care in Latvia, it was valuable to evaluate its effect.

Consistent with other studies [31], we observed a lower antibiotic prescribing rate than expected. This may have been due to knowledge regarding the aim of the study influencing participants’ prescribing habits. However, this factor would be expected to affect both study groups equally.

To reflect real daily practice, we asked the FP not to use POCT for all patients but only for those they were unsure about prescribing antibiotics for and felt that additional testing would help their decision-making. This is in line with other studies where testing was not recommended for all patients but restricted to those believed to be at higher risk following clinical assessment [21]. Previous studies have shown that for patient visits where FP considered CRP testing crucial, the result influenced their decision on antibiotic prescribing more often [25]. Furthermore, gratuitous testing could even increase antibiotic prescribing [31, 32]. Nevertheless, the FP ordered CRP testing frequently, possibly because the test was not accessible in their practice prior to the study and they were interested in trying it.

A limitation of this study is that FP did not include all patients with acute infection episodes who visited FP during the study period, but still the target number of patients was achieved. Also, we don’t have follow up data of patient’s recovery, hospitalization or subsequent antibiotic prescribing.

## Conclusion

Our results show that the availabilty of CRP POCT and educational training for FP did not reduce antibiotic prescribing. Nevertheless, our data indicate that regional variations in antibiotic-prescribing habits exist and the implemented interventions had an effect on FP practices in rural areas.

In the absence of CRP POCT, especially in rural areas, patients undergo minimal CRP testing prior to antibiotic prescribing, consequently leading to initiation of unwarranted antibacterial treatment.

## Data Availability

The data presented in this study can be viewed on a separate dataset spreadsheet: https://docs.google.com/spreadsheets/d/1KqPJRWe9Ib15_mwHYbERLcLvbYett6VG.

## Abbreviations

FP: Family physicians
POCT: Point-of-care testing
CRP: C-reactive protein
AB: Antibiotic

## References

[1] Pottegård A, Broe A, Aabenhus R, Bjerrum L, Hallas J, Damkier P (2015). Use of antibiotics in children: a Danish nationwide drug utilization study. Pediatr Infect Dis J.

[2] Dekker ARJ, Verheij TJM, Van Der Velden AW (2017). Antibiotic management of children with infectious diseases in Dutch Primary Care. Fam Pract.

[3] King LM, Fleming-Dutra KE, Hicks LA (2018). Advances in optimizing the prescription of antibiotics in outpatient settings. BMJ.

[4] Schrier L, Hadjipanayis A, del Torso S, Stiris T, Emonts M, Dornbusch HJ (2018). European Antibiotic Awareness Day 2017: training the next generation of health care professionals in antibiotic stewardship. Eur J Pediatr.

[5] van Houten CB, Cohen A, Engelhard D, Hays JP, Karlsson R, Moore E (2019). Antibiotic misuse in respiratory tract infections in children and adults—a prospective, multicentre study (TAILORED treatment). Eur J Clin Microbiol Infect Dis.

[6] van der Velden AW, Pijpers EJ, Kuyvenhoven MM, Tonkin-Crine SK, Little P, Verheij TJ (2012). Effectiveness of physician-targeted interventions to improve antibiotic use for respiratory tract infections. Br J Gen Pract.

[7] Arnold S, Straus S (2006). Interventions to improve antibiotic prescribing practices in ambulatory care. Evid Based Child Heal A Cochrane Rev J.

[8] Cooke J, Butler C, Hopstaken R, Dryden MS, Mcnulty C, Hurding S, et al. Narrative review of primary care point-of-care testing (POCT) and antibacterial use in respiratory tract infection (RTI); Available from: http://www. Cited 2021 Feb 9. 10.1136/bmjresp-2015-000086 PMC4426285 25973210

[9] Kip MMA, Hummel JM, Eppink EB, Koffijberg H, Hopstaken RM, IJzerman MJ (2019). Understanding the adoption and use of point-of-care tests in Dutch general practices using multi-criteria decision analysis. BMC Fam Pract.

[10] Huang Y, Chen R, Wu T, Wei X, Guo A (2013). Association between point-of-care CRP testing and antibiotic prescribing in respiratory tract infections: a systematic review and meta-analysis of primary care studies. Br J Gen Pract.

[11] Van Hecke O, Raymond M, Lee JJ, Turner P, Goyder CR, Verbakel JY, et al. In-vitro diagnostic point-of-care tests in paediatric ambulatory care: a systematic review and meta-analysis. Plos One. 2020;15(7) Available from:. 10.1371/journal.pone.0235605. 10.1371/journal.pone.0235605 PMC7337322 32628707

[12] Principi N, Esposito S (2016). Antimicrobial stewardship in paediatrics.

[13] Urbiztondo I, Bjerrum L, Caballero L, Suarez MA, Olinisky M, Córdoba G. Decreasing inappropriate use of antibiotics in primary care in four countries in South America—cluster randomized controlled trial. Antibiotics. 2017;6(4) Available from: https://www.ncbi.nlm.nih.gov/pmc/articles/PMC5745481/. Cited 2021 Sep 28. 10.3390/antibiotics6040038 PMC5745481 29240687

[14] Llor C, Bjerrum L, Molero JM, Moragas A, López-Valcárcel BG, Monedero MJ (2018). Long-term effect of a practice-based intervention (HAPPY AUDIT) aimed at reducing antibiotic prescribing in patients with respiratory tract infections. J Antimicrob Chemother.

[15] Llor C, Cots JM, Hernández S, Ortega J, Arranz J, Monedero MJ (2014). Effectiveness of two types of intervention on antibiotic prescribing in respiratory tract infections in Primary Care in Spain. Happy Audit Study. Aten Primaria.

[16] Likopa Z, Kivite-Urtane A, Pavare J. Latvian primary care management of children with acute infections: antibiotic-prescribing habits and diagnostic process prior to treatment. Medicina (B Aires). 2021;57(8) Available from: https://www.ncbi.nlm.nih.gov/pmc/articles/PMC8397978/. Cited 2021 Dec 20. 10.3390/medicina57080831 PMC8397978 34441037

[17] Lemiengre MB, Verbakel JY, Colman R, De Burghgraeve T, Buntinx F, Aertgeerts B (2018). Reducing inappropriate antibiotic prescribing for children in primary care: a cluster randomised controlled trial of two interventions. Br J Gen Pract.

[18] Martínez-González NA, Keizer E, Plate A, Coenen S, Valeri F, Verbakel JYJ, et al. Point-of-care c-reactive protein testing to reduce antibiotic prescribing for respiratory tract infections in primary care: systematic review and meta-analysis of randomised controlled trials. Antibiotics. 2020;9:1–31 MDPI AG; Available from: /pmc/articles/PMC7559694/?report=abstract. Cited 2020 Nov 23. 10.3390/antibiotics9090610 PMC7559694 32948060

[19] Fleiss JL, Levin B, Paik MC (2003). Statistical Methods for Rates and Proportions.

[20] Althaus T, Greer RC, Swe MMM, Cohen J, Tun NN, Heaton J (2019). Effect of point-of-care C-reactive protein testing on antibiotic prescription in febrile patients attending primary care in Thailand and Myanmar: an open-label, randomised, controlled trial. Lancet Glob Heal.

[21] Verbakel JY, Lemiengre MB, De Burghgraeve T, De Sutter A, Aertgeerts B, Shinkins B (2016). Should all acutely ill children in primary care be tested with point-of-care CRP: a cluster randomised trial.

[22] Do NTT, Ta NTD, Tran NTH, Than HM, Vu BTN, Hoang LB (2016). Point-of-care C-reactive protein testing to reduce inappropriate use of antibiotics for non-severe acute respiratory infections in Vietnamese primary health care: a randomised controlled trial. Lancet Glob Heal.

[23] J Dekker AR, M Verheij TJ, L Broekhuizen BD, Butler CC, L Cals JW, Francis NA, et al. Effectiveness of general practitioner online training and an information booklet for parents on antibiotic prescribing for children with respiratory tract infection in primary care: a cluster randomized controlled trial; Available from: https://academic.oup.com/jac/article/73/5/1416/4846902. Cited 2021 Oct 19 10.1093/jac/dkx542 29438547

[24] Wei X, Zhang Z, Walley JD, Hicks JP, Zeng J, Deng S (2017). Articles Effect of a training and educational intervention for physicians and caregivers on antibiotic prescribing for upper respiratory tract infections in children at primary care facilities in rural China: a cluster-randomised controlled trial.

[25] Lindström J, Nordeman L, Hagström B (2015). What a difference a CRP makes. A prospective observational study on how point-of-care C-reactive protein testing influences antibiotic prescription for respiratory tract infections in Swedish primary health care. Scand J Prim Health Care.

[26] Dumpis U, Hahlin A, Varvuolyte S, Stenmark S, Veide S, Valinteliene R (2018). Antibiotic prescription and clinical management of common infections among general practitioners in Latvia, Lithuania, and Sweden: a pilot survey with a simple protocol. Eur J Clin Microbiol Infect Dis.

[27] Curtis HJ, Walker AJ, Mahtani KR, Goldacre B (2019). Time trends and geographical variation in prescribing of antibiotics in England 1998-2017. J Antimicrob Chemother.

[28] Yau JW, Thor SM, Tsai D, Speare T, Rissel C (2021). Antimicrobial stewardship in rural and remote primary health care: a narrative review. Antimicrob Resist Infect Control.

[29] Xue H, Shi Y, Huang L, Yi H, Zhou H, Zhou C (2019). Diagnostic ability and inappropriate antibiotic prescriptions: a quasi-experimental study of primary care providers in rural China. J Antimicrob Chemother.

[30] Cals JWL, Butler CC, Hopstaken RM, Hood K, Dinant GJ (2009). Effect of point of care testing for C reactive protein and training in communication skills on antibiotic use in lower respiratory tract infections: cluster randomised trial. BMJ.

[31] Lemiengre MB, Verbakel JY, Colman R, Van Roy K, De Burghgraeve T, Buntinx F (2018). Point-of-care CRP matters: normal CRP levels reduce immediate antibiotic prescribing for acutely ill children in primary care: a cluster randomized controlled trial. Scand J Prim Health Care.

[32] Rebnord IK, Sandvik H, Batman Mjelle A, Hunskaar S (2016). Out-of-hours antibiotic prescription after screening with C reactive protein: a randomised controlled study. BMJ Open.

